# Sleep conditions and sleep hygiene behaviors in early pregnancy are associated with gestational diabetes mellitus: A propensity-score matched study

**DOI:** 10.1007/s11325-024-03071-8

**Published:** 2024-08-27

**Authors:** Guojun Ma, Yanqing Cai, Yong Zhang, Jianxia Fan

**Affiliations:** 1 School of Medicine, The International Peace Maternity and Child Health Hospital, Shanghai Jiao Tong University, Shanghai, China; 2 Shanghai Key Laboratory of Embryo Original Diseases, Shanghai, China; 3 Research Units of Embryo Original Diseases, Chinese Academy of Medical Sciences, Shanghai, China; 4 Department of Obstetrics and Gynecology, School of Medicine, The International Peace Maternity and Child Health Hospital, Shanghai Jiao Tong University, 910 Hengshan Road, Shanghai, 200030 China

**Keywords:** First pregnancy trimester, Gestational diabetes mellitus, Sleep hygiene, Sleep quality, Sleepiness

## Abstract

**Purpose:**

To explore the influence of sleep conditions and sleep hygiene behaviors in early pregnancy on gestational diabetes mellitus (GDM) development.

**Methods:**

This 1:1 propensity-score matched study included 1,216 pregnant women divided into GDM and control groups based on diagnosis via the oral glucose tolerance test at 24–28 gestational weeks. Sleep conditions and hygiene behaviors were evaluated using structural questionnaires, including the Pittsburgh Sleep Quality Index, Epworth Sleepiness Scale, and Sleep Hygiene Practice Scale. Univariate and multivariate logistic regression analyses and Spearman’s correlation were conducted to identify the associations.

**Results:**

After adjusting for baseline clinical characteristics, women with GDM were more likely to have poor sleep quality (adjusted odds ratio [AOR] = 1.585, 95% confidence interval [CI]: 1.261–1.992) and higher scores for subjective sleep quality, latency, duration, efficiency, and sleep disturbances (all *P* < 0.01). Mild sleepiness (AOR = 1.311, 95% CI: 1.012–1.699) and worrying about not being able to fall asleep (AOR = 1.123, 95% CI: 1.005–1.255) were more likely to occur in the GDM group. Sleep quality and hygiene behaviors such as sleep-irrelevant activities, staying in bed after waking up, weekend catch-up sleep, and overeating before bedtime were significantly correlated with gestational diabetes variables.

**Conclusion:**

Poor sleep conditions and specific sleep hygiene behaviors in early pregnancy may be independent risk factors for GDM. This suggests that sleep assessment and behavior education can be used as new approaches for the early implementation of surveillance and prevention of GDM.

**Supplementary Information:**

The online version contains supplementary material available at 10.1007/s11325-024-03071-8.

## Introduction

Over the past 20 years, the prevalence of gestational diabetes mellitus (GDM) has increased by more than 30%. In contrast to Europe and America, where the incidence is only 5–8%, the incidence of GDM in China is as high as 19.7% [1, 2], which has a significant impact on the short- and long-term outcomes of mothers and infants. These include pregnancy induced hypertension, caesarean delivery, preterm birth, neonatal hypoglycemia, intrauterine growth delay, as well as type 2 diabetes mellitus, obesity, and metabolic syndrome in mothers and the offspring [3, 4]. Therefore, researchers hope to improve the prognosis of mothers and infants by studying the etiology and risk factors of gestational diabetes.

Poor sleep quality is associated with a range of adverse health outcomes, including metabolic diseases, depression, anxiety, and neuromuscular disease [5]. Many studies have confirmed that sleep disorders are closely related to gestational diabetes [6]. According to our preliminary study, the proportion of pregnant women with poor sleep quality in early pregnancy was as high as 45.7%. The risk of GDM complications was significantly higher than that of pregnant women with good sleep quality (odds ratio [OR] = 1.573, 95% confidence interval [CI] 1.315–1.863) [7]. Sleep quality and duration directly affect endocrine, metabolic, and nervous system functions in pregnant women, leading to various adverse obstetric outcomes [8–10].

Sleep evaluation is divided into two main categories: subjective and objective. The former typically employs questionnaires that assess multiple dimensions related to sleep, including sleep quality, alertness/sleepiness, and sleep hygiene behaviors, whereas the latter employs more objective methods [11, 12]. Many reports have focused on the relationship between sleep hygiene habits and sleep quality; however, few have focused on the period during pregnancy [13–15]. Most studies have focused on the effects of sleep deprivation in the second and third trimesters of pregnancy with GDM [16–18], whereas few studies have focused on sleep status in the first stages of pregnancy. However, studies from the perspective of pregnancy physiology have found that maternal sleep patterns change significantly at 11–12 weeks of gestation compared with pre-pregnancy sleep characteristics [19]. This study aimed to understand the risk factors for GDM associated with sleep conditions and unhealthy sleep hygiene behaviors. Strengthening sleep management in pregnant women during early pregnancy is important to prevent the occurrence of GDM.

## Methods

### Study population

This prospective, single-center, 1:1 matched case-control study was performed at the International Peace Maternity and Child Health Hospital (IPMCH) between June 2020 and June 2021 in Shanghai, China. Pregnant women who underwent the first prenatal examination before 13 weeks of gestation at the IPMCH and provided valid and completed questionnaires were included in the study (*n* = 3,000).

The exclusion criteria were as follows: (1) age < 18 or > 45 years; (2) inability to complete the relevant investigation due to mental or communication barriers; (3) long-term use of psychotropic drugs, antidepressants, and sedative hypnotic drugs; (4) multiple pregnancies; (5) missing information on birth outcomes; (6) history of GDM or preterm birth (< 37 weeks); and (7) preexisting chronic diseases, such as diabetes or hypertension before pregnancy.

Data were obtained from the IPMCH electronic medical record system. All the participants signed an informed consent form. This study was approved by the Ethics Committee of IPMCH (GKLW 2019-58).

### Data collection

At the first prenatal visit, pregnant women were questioned by educated investigators regarding general and sleep information using a structured questionnaire after obtaining informed consent. The study investigator guided the pregnant women to complete the questionnaires alone; they then collected the questionnaires and checked for completion. A total of 3,000 questionnaires were distributed, with an effective recovery rate of 100%. A total of 2,703 valid questionnaires with complete data were delivered to our hospital, with a loss rate of 9.83%.

Baseline clinical information included maternal age, maternal education level, pre-pregnancy body mass index (pre-BMI), parity, assisted reproductive technology (ART), and monthly family income. Pre-BMI was calculated using the following formula:

(1) BMI = *weight* (kg)/*height* (m^2^).

The outcome/dependent variable was GDM.

Relative sleep variables were assessed using three sleep assessment questionnaires (the Pittsburgh Sleep Quality Index [PSQI], Epworth Sleepiness Scale [ESS], and Sleep Hygiene Practice Scale [SHPS]). After completion, the questionnaires were scored using a unified evaluation formula to classify sleep quality, alertness/sleepiness, and sleep hygiene behaviors.

### Screening tools and diagnostic criteria

#### Subjective Sleep Quality Scale

The PSQI is the most commonly used tool for sleep quality screening worldwide. The scale comprises 19 items that pertain to an individual’s sleep over the previous month, and it is divided into seven subscales: subjective sleep quality, sleep latency, sleep duration, sleep efficiency, sleep disturbances, use of hypnotic medication, and daytime dysfunction [20]. Each subscale is scored on a scale from 0 to 3; the total score ranges from 0 to 21. Higher PSQI scores indicate poorer sleep quality, with a cutoff value of 5 points. A total score > 5 indicated poor sleep quality, while a score of 0–5 indicated good sleep quality. The validity of the Chinese PSQI was verified using PSQI scale tests. The sensitivity and specificity of the questionnaire were 98.0% and 55.0%, respectively, demonstrating that the Chinese PSQI scale has satisfactory reliability and validity [21].

The ESS is used to assess maternal arousal/drowsiness during pregnancy. The ESS consists of 8 items, with each item ranging from 0 to 3 points. The higher the score, the greater the sleepiness level. The conventional cutoff for excessive sleepiness is drawn at > 10 points; generally, ≤ 6 points indicates no sleepiness, and 7–10 points indicates mild sleepiness. This study used the Chinese version of the ESS that was translated and validated by Chen et al. [22].

The SHPS is used to assess sleep hygiene awareness and habits. The SHPS has a 30-item scale that measures four domains of sleep hygiene: domain 1, arousal-related behaviors; domain 2, sleep scheduling and timing; domain 3, eating/drinking behaviors; and domain 4, sleep environment [23]. Respondents rated each item on a 6-point Likert scale ranging from 1 (never) to 6 (always), and the total scale score ranged from 30 to 180. Higher total scores for each domain indicate greater maladaptive sleep hygiene. The Chinese version of SHPS showed acceptable test-retest reliability (Cronbach’s α coefficient, 0.880) [24]. The Cronbach’s α in the current study was 0.860.

### Diagnosis of GDM

According to the American Diabetes Association (ADA) criteria, GDM was diagnosed at 24–28 gestational weeks according to the plasma glucose levels during the 2 h, 75 g oral glucose tolerance test (OGTT). Abnormal values were defined according to the ADA thresholds: a fasting glucose value ≥ 5.1 mmol/L, a 1 h value ≥ 10.0 mmol/L, and a 2 h value ≥ 8.5 mmol/L.

### Chinese version of the Profile of Mood States (a-POMS)

The Profile of Mood States (a-POMS) is a questionnaire comprising seven components of mood: Depression-Dejection, Anger-Hostilities, Tension-Anxiety, Fatigue-Inertia, Vigor-Activity, Confusion-Bewilderment and Self-Esteem. The questionnaire comprises 40 items, which are accessed via a four-point Likert-type scale. A higher score for negative mood indicates a higher degree of mood disturbance. The score for total mood disturbance (TMD) was calculated as follows: score of (tension + anger + depression + confusion + fatigue) − score of (esteem-related affect + vigor) + 100 [25]. The construct validity and high internal consistency have been demonstrated in previous studies [26].

### Laboratory results

Fasting blood glucose (FBG), 1-hour blood glucose (1 h BG), 2-hour blood glucose (2 h BG), and glycated hemoglobin A1c (HbA1c) levels were measured using an automatic chemistry analyzer (BeckmanAU5800, Beckman, Brea, CA, USA). FBG and HbA1c levels were measured after overnight fasting during the first trimester (8–14 weeks of gestation) and OGTT (24–28 weeks of gestation).

### Statistical analysis

A 1:1 propensity-score matched (PSM) study was conducted to identify the remaining women without GDM with similar baseline characteristics, including maternal age (mean ± standard deviation [SD]; distribution: <30, 30–34, 35–39, ≧40 years), pre-BMI (mean ± SD; distribution: <18.5, 18.5–24, 24.1–28, > 28 kg/m^2^), maternal education level (high school or below, bachelor, master or above), parity (primiparous, multiparous), ART (no, yes), and monthly family income (< 10, 10–20, 20–30, > 30 thousands yuan). All variables were entered into a multivariate logistic regression model to calculate propensity scores, and matching was performed using a 1:1 nearest neighbor matching algorithm with a caliper width of 0.05 without replacement. A standardized mean difference of < 20% and a *P*-value greater than 0.05 indicated a relatively small imbalance.

The Kolmogorov–Smirnov test was used to evaluate the normal distribution. Normally distributed variables were expressed as mean ± SD, while non-normally distributed variables are expressed as median (interquartile range). Categorical variables were expressed as frequency (n) and percentages (%). Continuous variables were assessed using the Student’s *t*-test or the Mann–Whitney U test. Categorical data were analyzed using the chi-squared test or Fisher’s exact test. To identify the association between GDM, sleep conditions, sleep hygiene behaviors and mood states, univariate and multivariate logistic regression analyses were performed. ORs and 95% CIs were adjusted for all variables involved in PSM. Spearman’s correlation was used to evaluate the relationship between sleep conditions, sleep hygiene behaviors, and gestational diabetes variables. Statistical significance was defined as a two-sided *P*-value < 0.05. Statistical analysis was performed using the Statistical Product and Service Solutions (version 24.0, SPSS, Chicago, IL, USA) and R statistical software version 4.2.3 (packages “MatchIt,” “tableone,” “forestploter,” “psych,” “ggplot2,” and “pheatmap”).

## Results

### Baseline clinical characteristics

**Fig. 1:**
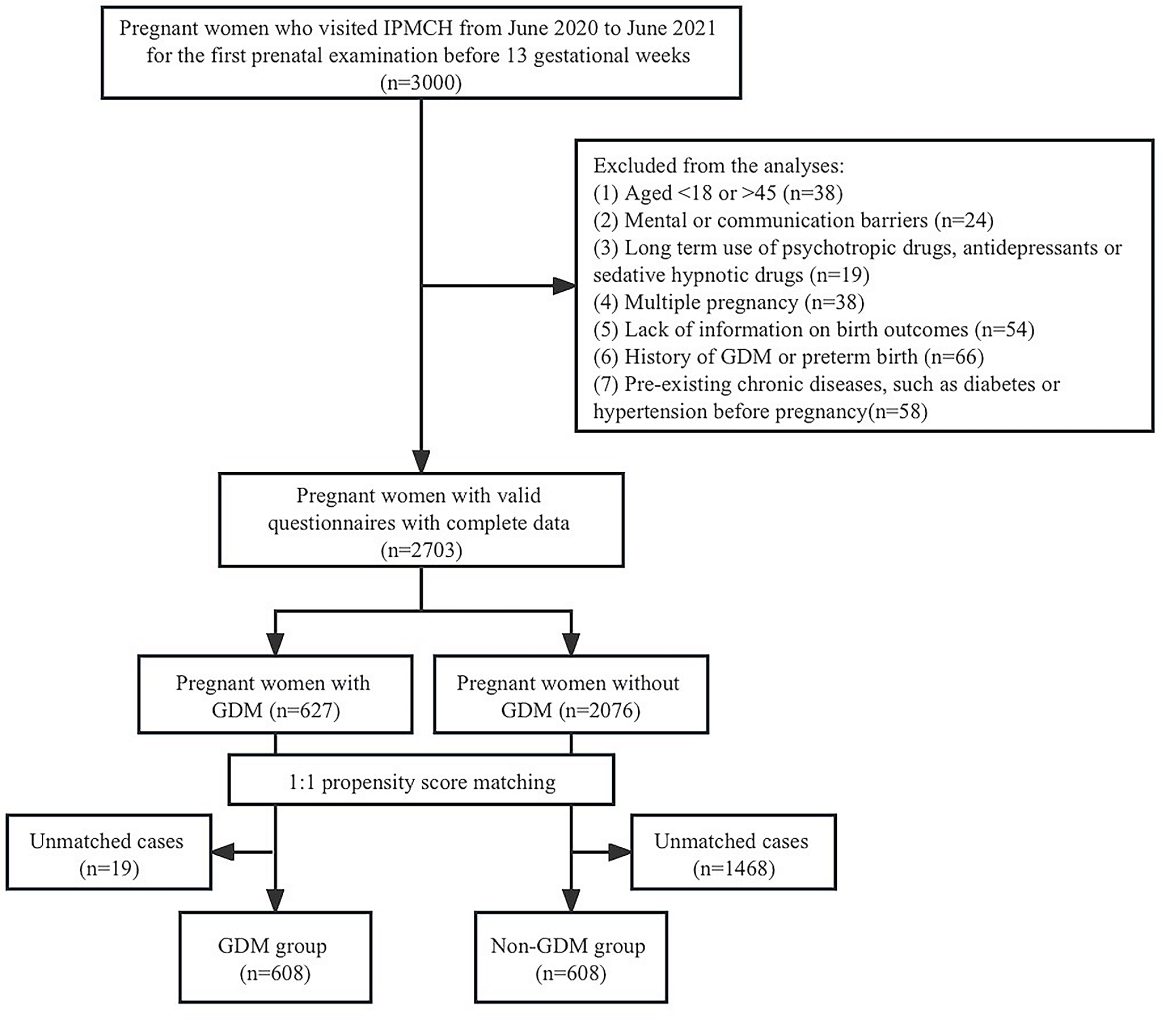
Flow Chart

A total of 3,000 women completed valid questionnaires during the study period. Among them, 297 were excluded according to the criteria, and the remaining were further classified as having GDM (*n* = 627) or not having GDM (*n* = 2,076). Finally, we constructed a PSM population comprising 608 women in each group (flow chart shown in Fig. 1).

Comparisons of the clinical characteristics of the two groups before and after PSM are shown in Table 1. Before PSM, the maternal age (31.84 ± 3.967 vs. 30.68 ± 3.683, *P* < 0.001), pre-BMI (22.31 ± 3.162 vs. 20.98 ± 4.113, *P* < 0.001), and the proportion of women with ART (12.6% vs. 8.6%, *P* = 0.004) in the GDM group were higher than that in the control group. The proportion of women with a master’s degree or above was lower in the GDM group than in the control group (17.9% vs. 23.6%, *P* = 0.010). After matching, there were no significant differences between the two groups.

**Table 1:** Baseline clinical characteristics of participants before and after propensity-score matching

	Before Matching					After Matching			
	Non-GDM group *n* = 2076	GDM group *n* = 627	SMD	*P*-value		Non-GDM group *n* = 608	GDM group *n* = 608	SMD	*P*-value
**Maternal characteristics**									
Maternal age (year)									
Mean ± SD	30.68 ± 3.683	31.84 ± 3.967	0.302	< 0.001		31.80 ± 3.792	31.79 ± 3.983	0.002	0.976
Distribution			0.277	< 0.001				0.085	0.538
< 30	1076(51.8)	254(40.5)				241(39.6)	250(41.1)		
30–34	680(32.8)	225(35.9)				220(36.2)	216(35.5)		
35–39	297(14.3)	126(20.1)				133(21.9)	121(19.9)		
≧ 40	23(1.1)	22(3.5)				14(2.3)	21(3.5)		
Pre-BMI (kg/m^2^)									
Mean ± SD	20.98 ± 4.113	22.31 ± 3.162	0.364	< 0.001		21.86 ± 2.861	22.07 ± 2.838	0.075	0.188
Distribution			0.444	< 0.001				0.064	0.739
< 18.5	316(15.2)	40(6.4)				38(6.2)	40(6.6)		
18.5–24	1532(73.8)	431(68.7)				430(70.7)	431(70.9)		
24.1–28	193(9.3)	121(19.3)				115(18.9)	119(19.6)		
> 28	35(1.7)	35(5.6)				25(4.1)	18(3.0)		
Parity			0.061	0.179				0.058	0.340
Primiparous	1581(76.2)	461(73.5)				427(70.2)	443(72.9)		
Multiparous	495(23.8)	166(26.5)				181(29.8)	165(27.1)		
ART			0.129	0.004				0.026	0.722
No	1897(91.4)	548(87.4)				539(88.7)	534(87.8)		
Yes	179(8.6)	79(12.6)				69(11.3)	74(12.2)		
**Socio-demographic characteristics**									
Monthly income of the family (thousands yuan)			0.085	0.307				0.147	0.087
< 10	143(6.9)	55(8.8)				31(5.1)	53(8.7)		
10–20	843(40.6)	242(38.6)				250(41.1)	233(38.3)		
20–30	641(30.9)	185(29.5)				190(31.2)	182(29.9)		
> 30	449(21.6)	145(23.1)				137(22.5)	140(23.0)		
Maternal education level			0.142	0.010				0.049	0.691
High school or below	152(7.3)	53(8.5)				47(7.7)	51(8.4)		
Bachelor	1435(69.1)	462(73.7)				439(72.2)	446(73.4)		
Master or above	489(23.6)	112(17.9)				122(20.1)	111(18.3)		

Data was presented as mean ± SD or frequency (percentage)

Abbreviations: GDM, gestational diabetes mellitus; Pre-BMI, pre-pregnancy body mass index; ART, assisted reproductive technology; SD, standard deviation; SMD, standardized mean difference

### Association between sleep conditions and GDM

**Fig. 2:**
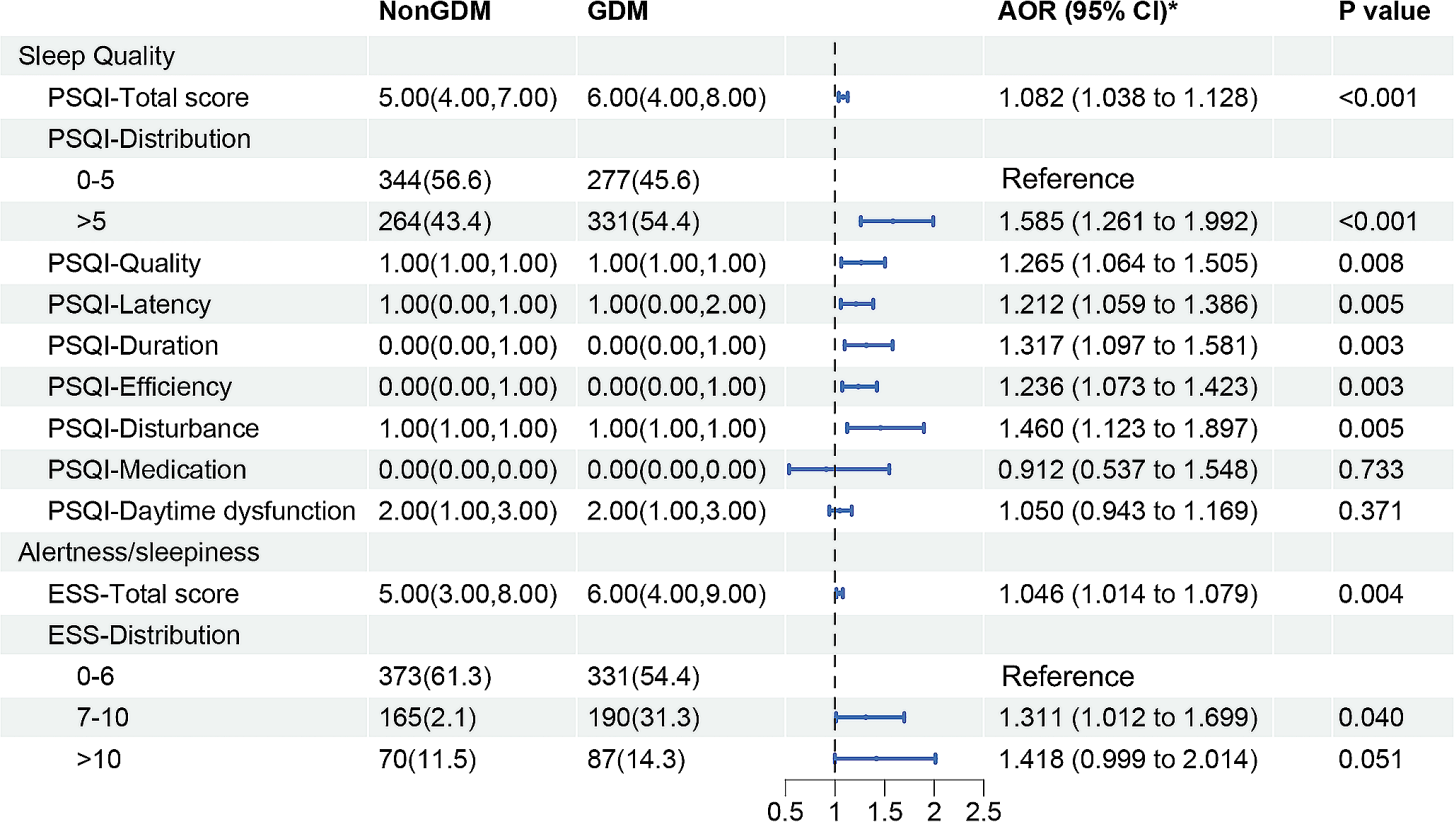
Sleep conditions of participants in the propensity-score matched cohort. Data was presented as median (interquartile range, IQR) or frequency (percentage). ^*^Adjusted for all baseline clinical factors mentioned in PSM. Abbreviations: GDM, gestational diabetes mellitus; PSQI, the Pittsburgh Sleep Quality Index; ESS, the Epworth Sleepiness Scale; AOR, adjusted odds ratio; CI, confidence interval

Univariate and multivariate logistic regression analyses were used to evaluate sleep conditions **(**Fig. 2, **Online Resource 1)**. Compared with the control group, women in the GDM group were more likely to have poor sleep quality (total PSQI score > 5) (54.4% vs. 43.4%, adjusted OR [AOR] = 1.585, 95% CI: 1.261–1.992, *P* < 0.001). The results also showed that the PSQI total score and some of the subscales (subjective sleep quality, sleep latency, sleep duration, sleep efficiency, and sleep disturbances) of participants without GDM were significantly lower than those of participants with GDM (all *P* < 0.01). No significant differences in hypnotic medication and daytime dysfunction scores between the two groups were observed.

Regarding alertness/sleepiness, the participants without GDM had lower ESS-total scores than those with GDM (5.00 [3.00, 8.00] vs. 6.00 [4.00, 9.00], *P* = 0.004). Women in the GDM group were more likely to have mild sleepiness (total ESS score 7–10) (31.3% vs. 2.1%, AOR = 1.311, 95% CI: 1.012–1.699, *P* = 0.040). However, the proportion of women with excessive sleepiness (total ESS score > 10) in the GDM group was not significantly higher than that in the control group (14.3% vs. 11.5%, AOR = 1.418, 95% CI: 0.999–2.014, *P* = 0.051).

### Association between sleep hygiene behaviors and GDM

**Fig. 3:**
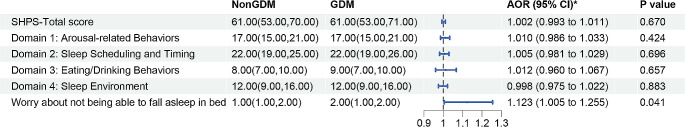
Sleep hygiene behaviors of participants in the propensity-score matched cohort. Data was presented as median (interquartile range, IQR). ^*^Adjusted for all baseline clinical factors mentioned in PSM. Abbreviations: GDM, gestational diabetes mellitus; SHPS, the Sleep Hygiene Practice Scale; AOR, adjusted odds ratio; CI, confidence interval

After adjusting for all clinical characteristics mentioned in the PSM, we found that GDM was associated with worrying about not being able to fall asleep (AOR = 1.123, 95% CI: 1.005–1.255, *P* = 0.041). No differences were found between the two groups in the total SHPS score, the four domains, or the remaining behaviors (Fig. 3, **Online Resource 2)**.

### Correlations between sleep conditions and sleep hygiene behaviors with gestational diabetes variables

**Fig. 4:**
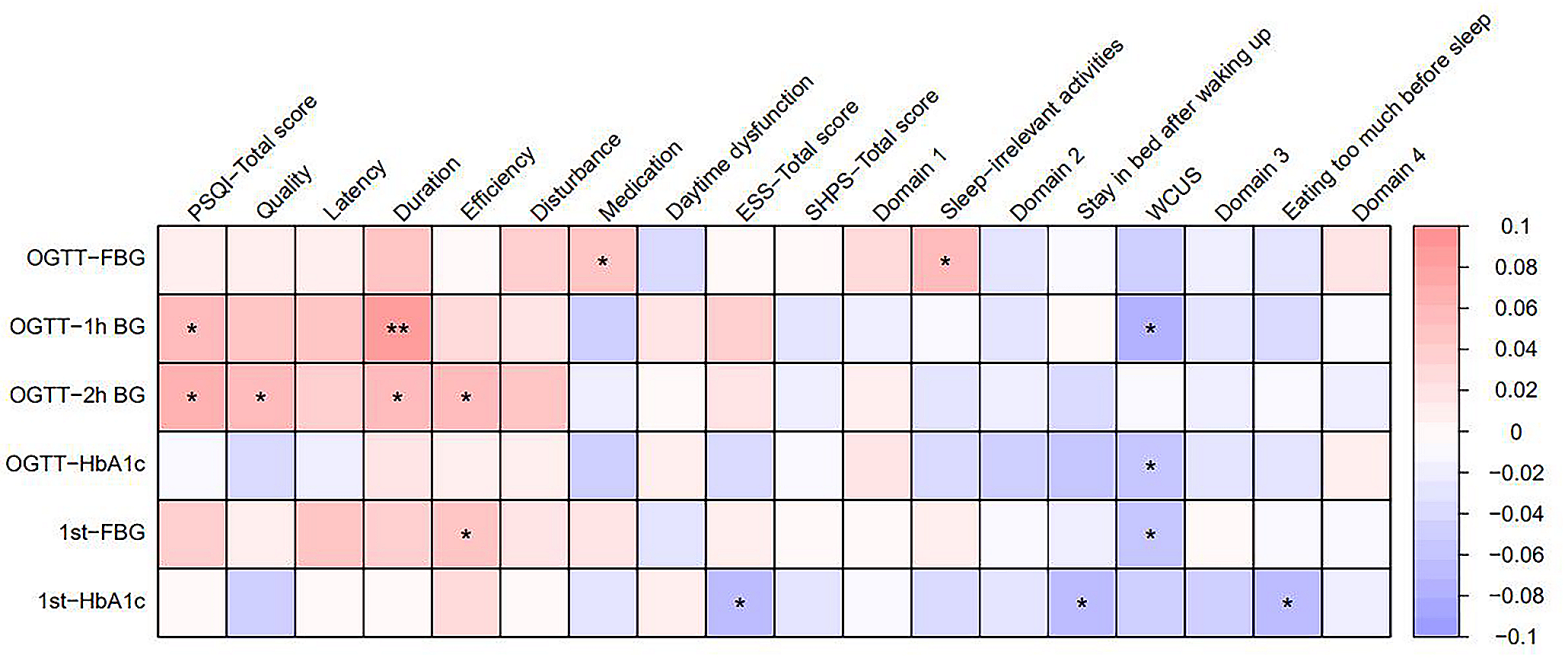
Correlations between sleep conditions and sleep hygiene behaviors with gestational diabetes variables. ^*^P < 0.05, ^**^P < 0.01. Abbreviations: OGTT, oral glucose tolerance test; 1st, the first trimester of pregnancy; FBG, fasting blood glucose; 1h BG, 1-hour blood glucose; 2h BG, 2-hour blood glucose; HbA1c, glycated hemoglobin A1c; PSQI, the Pittsburgh Sleep Quality Index; ESS, the Epworth Sleepiness Scale; SHPS, the Sleep Hygiene Practice Scale; WCUS, weekend catch-up sleep

Spearman’s correlations for all sleep and gestational diabetes variables are shown in **Online Resource 3**. In addition, sleep conditions and efficient individual items of sleep hygiene behaviors were selected to construct a heatmap (Fig. 4). The results showed that the correlations between most of the key scores regarding sleep conditions, including PSQI-Total score, PSQI-Quality, PSQI-Duration, PSQI-Efficiency, PSQI-Medication, and OGTT-BG results were positively significant (*P* < 0.05). Furthermore, the ESS-Total score and first trimester HbA1c level correlated negatively (*r* = -0.064, *P* = 0.029).

However, the SHPS-Total score and the four domains were not significantly associated with the gestational diabetes results (all *P* > 0.05). Several arousal-related and sleep-scheduling hygiene behaviors correlated significantly with the OGTT results, including sleep-irrelevant activities with OGTT-FBG (*r* = 0.068, *P* = 0.017) and weekend catch-up sleep (WCUS) with OGTT-1 h BG (*r* = -0.073, *P* = 0.011) and OGTT-HbA1c (*r* = -0.060, *P* = 0.038). In the first trimester, sleep scheduling and eating/drinking behaviors correlated significantly with laboratory results.

### Association between mood states varieties and GDM

The GDM was associated with POMS-Tension (AOR = 1.033, 95% CI: 1.005–1.063, *P* = 0.021) after adjusting for all clinical characteristics mentioned in the PSM. There were no significant differences between the two groups with respect to TMD and other mood states (Online Resource 4).

## Discussion

### The impact of maternal sleep quality and sleepiness on GDM

In the present study, we found that the prevalence of GDM was as high as 23.2% (627/2,703). After PSM and adjustment for various baseline clinical characteristics, we found that most key sleep quality variables were significantly associated with the incidence of GDM and positively correlated with the OGTT results. These findings were consistent with those of previous studies. A longitudinal study of 4,550 Chinese women showed that a one-point PSQI score increase was associated with a 0.014 mmol/L increase in the blood glucose level [27]. Gooley reviewed five studies and summarized that the frequency of GDM is higher in women with a shorter sleep duration than in those with a longer sleep duration [28]. In studies on sleep deprivation, it has been demonstrated short sleep duration and decreased slow wave sleep (SWS) may amplify the inflammatory response [29], decrease sympathetic activity, cause dysregulation of the hypothalamic-pituitary-adrenal axis and cortisol secretion, and stimulate growth hormone release [30, 31], all of which are likely to decrease downstream insulin signaling and insulin sensitivity. Additionally, recent research indicates that circadian misalignment and insufficient sleep may affect appetite hormones, including elevating ghrelin levels and reducing leptin sensitivity [32]. Consequently, there is a biologically plausible link between poor sleep quality and the risk of GDM.

In addition, the multivariate analysis showed that the total ESS score and mild sleepiness were significant risk factors for GDM. Our previous studies demonstrated that sleep quality evaluated using the PSQI score positively correlated with sleepiness evaluated using the ESS (*r* = 0.184, *P* < 0.01). Previous studies have shown that excessive sleepiness affects the daytime functioning of pregnant women [33, 34]. This further indicates that the ESS can effectively evaluate the sleep status of pregnant women and guide doctors in detecting and preventing the occurrence of GDM during early pregnancy.

### The impact of maternal sleep hygiene behaviors on GDM

Worse sleep hygiene behaviors can lead to significantly lower sleep efficiency in pregnant women, often manifesting as shorter sleep duration and longer wake time [15, 35]. In our study, arousal-related and sleep-scheduling behaviors played important roles in GDM and blood glucose elevation.

Arousal behaviors are defined as a series of sleep practices that enhance arousal by promoting anxiety and/or conditioned arousal [23]. We found that worrying about not being able to fall asleep was a unique predictor of GDM, and sleep-irrelevant activities in bed had a significant positive correlation with OGTT-FBG. Räikkönen et al. demonstrated that tension was a contributing factor in the development of metabolic syndrome in healthy middle-aged women [36]. A previous study conducted by our research team revealed a correlation between tension during early pregnancy and poor sleep quality [7]. Combined with the current study, it can be postulated that tension during early pregnancy influences sleep quality and metabolism, which may contribute to GDM. We speculate that the higher risk of GDM among pregnant women with arousal-related behaviors and anxiety states can result in insulin resistance and impaired glucose homeostasis by triggering inflammatory responses and oxidative stress. Tsai et al. found that pre-sleep arousal behaviors were significantly associated with poor sleep quality in third-trimester pregnant women [35]. Safety behaviors aimed at avoiding poor sleep consequences, such as worrying about sleep capacity and checking time, may induce more awakenings and create a vicious cycle that leads to poor sleep quality [37]. Thus, this vicious cycle may strengthen the link between GDM and arousal-related behaviors. These findings suggest that we should consider whether cognitive-behavioral strategies may trigger maternal anxiety or even exacerbate the occurrence of arousal.

Regarding sleep scheduling, WCUS and staying in bed after waking were negatively associated with gestational diabetes variables. These results support the beneficial effects of WCUS and longer time spent in bed in lowering the risk of GDM during early pregnancy. This can be explained by the effects of sufficient “recovery sleep” on stabilizing changes in carbohydrate metabolism (such as insulin-like growth factor-1 and myeloperoxidase) and improving the recovery process of immune and inflammatory parameters caused by lack of sleep [38, 39]. Longer sleep duration and time spent in bed on weekends and weekdays were significantly associated with better insulin sensitivity in adolescents with obesity [40]. Several studies have shown that WCUS is significantly associated with a lower prevalence of metabolic syndrome (AOR = 0.718, 95% CI: 0.554–0.931) [41] and is negatively and independently associated with BMI (*B* = -0.09, 95% CI: -0.11 to -0.06) [42]. Therefore, avoiding physiologically and emotionally arousing activities before bedtime and increasing sufficient “recovery sleep” might be a target for sleep-hygiene intervention in women during pregnancy.

### Suggestions on sleep management of women in early pregnancy

The following recommendations and intervention strategies for sleep during pregnancy are based on the findings of our study. First, the establishment of effective sleep reflexes and the reduction of tension can be highly beneficial in facilitating the process of falling asleep. It is crucial to acknowledge that attempting to resolve sleep issues through excessive worry is an ineffective approach. Only activities related to sleep should be conducted in the bedchamber; other activities (e.g., watching television, reading, playing with mobile phones, etc.) should be conducted in an alternative location. Second, an increase in the duration of sleep can enhance the quality of sleep and improve the efficiency of sleep. It is recommended that the individual continue to spend time in bed after waking up in the morning, and that they increase the amount of supplemental sleep on weekends. Third, when sleep difficulties arise, it is recommended that individuals engage in relaxation training techniques. These may include abdominal breathing exercises, muscle relaxation practices and mindfulness meditation, which have been demonstrated to be effective in improving sleep quality [43].

### Limitations

The strength of our study is that we used a selection of subjective methods in early pregnancy and completed relatively comprehensive assessments of different sleep dimensions. However, our study has several limitations. First, the potential limitations of subjective instruments for sleep conditions and sleep hygiene behaviors should be considered. Second, we did not collect follow-up data on sleep variables during the second and third trimesters; therefore, the long-term development of GDM could not be traced. As such, a longitudinal study design using subjective and objective sleep measures is required to explore the predictive relationships during pregnancy and establish generalizability. Third, more additional potential confounding factors which may influence glucose metabolism, including mood states, smoking and alcohol consumption prior to pregnancy, should be incorporated into the PSM procedure and controlled for.

In conclusion, we found that poor sleep quality, mild sleepiness, and arousal-related behaviors were related to GDM. This suggests that these factors can be tracked, allowing for early implementation of surveillance and prevention of GDM. Moreover, we suggest that sleep assessment and sleep behavior education in early pregnancy can be effective measures to prevent the development of gestational diabetes.

## Electronic supplementary material

Below is the link to the electronic supplementary material.

Supplementary Material 1

Supplementary Material 2

Supplementary Material 3

Supplementary Material 4

## Data Availability

The datasets are available from the corresponding author upon reasonable request.
