# Supplementary material for: Sleep conditions and sleep hygiene behaviors in early pregnancy are associated with gestational diabetes mellitus: A propensity-score matched study
Source: Sleep Breath. 2024 Aug 27;28(6):2421–30. doi: 10.1007/s11325-024-03071-8 (PMC11567980; doi:10.1007/s11325-024-03071-8)
Supplement: Supplementary file 1 — Supplementary Material 1 [file 11325_2024_3071_MOESM1_ESM.pdf]

Supplementary Information (SI)

Online Resource 3

Sleep conditions and sleep hygiene behaviors in early pregnancy are associated with gestational diabetes mellitus: A propensity-score matched study

Sleep and Breathing

Guojun Ma[1,2,3, a] · Yanqing Cai[1,2,3, a] · Yong Zhang[1,2,3,*] · Jianxia Fan[1,2,3,*]

Co-corresponding author: Yong Zhang and Jianxia Fan

Address correspondence to:

Dr. Jianxia Fan, Department of Obstetrics and Gynecology, the International Peace Maternity and Child Health Hospital, School of Medicine, Shanghai Jiao Tong University, 910 Hengshan Road, Shanghai, 200030, China. Tel: +8613916212979. E-mail address: fanjianxia122@126.com

Dr. Yong Zhang, Department of Obstetrics and Gynecology, the International Peace Maternity and Child Health Hospital, School of Medicine, Shanghai Jiao Tong University, 910 Hengshan Road, Shanghai, 200030, China. Tel: +8613916472189. E-mail address: yongz415@163.com

Online Resource 3 Correlations between sleep conditions and sleep hygiene behaviors with gestational diabetes variables

	OGTT				First trimester	
	FBG	1h BG	2h BG	HbA1c	FBG	HbA1c
**Sleep Conditions**						
PSQI-Total score	0.016	0.068*	0.071*	-0.008	0.042	0.003
PSQI-Quality	0.011	0.050	0.060*	-0.033	0.018	-0.040
PSQI-Latency	0.010	0.056	0.048	-0.015	0.057	0.004
PSQI-Duration	0.051	0.095**	0.061*	0.030	0.046	0.008
PSQI-Efficiency	0.009	0.039	0.068*	0.016	0.059*	0.032
PSQI-Disturbance	0.049	0.030	0.055	0.011	0.028	0.010
PSQI-Medication	0.058*	-0.042	-0.018	-0.045	0.022	-0.025
PSQI-Daytime dysfunction	-0.036	0.020	0.010	0.011	-0.021	0.016
ESS-Total score	0.006	0.046	0.024	-0.035	0.014	-0.064*
**Sleep Hygiene Behaviors**						
**SHPS-Total score**	0.008	-0.025	-0.011	-0.003	0.005	-0.022
**Domain 1: Arousal-related Behaviors**	0.034	-0.014	0.010	0.030	0.002	-0.001
Doing sleep-irrelevant activities in bed (e.g., watching TV, reading)	0.068*	-0.009	-0.023	-0.031	0.014	-0.035
Worry about not being able to fall asleep in bed	-0.004	0.009	0.027	0.037	-0.007	0.018
Unpleasant conversation prior to sleep	0.030	-0.016	0.003	0.032	0.027	-0.003
Not enough time to relax prior to sleep	0.027	0.003	0.012	0.042	-0.010	0.030
Falling asleep with TV or music on	0.025	0.005	0.020	0.024	-0.008	0.012
Pondering about unresolved matters while lying in bed	-0.007	-0.007	0.030	0.034	0.006	0.001
Check the time in the middle of night	0.000	0.011	0.022	0.024	0.023	0.010
Worry about night-time sleep during the day	-0.002	0.005	0.029	0.046	0.004	0.018
Vigorous exercise during the two hours prior to sleep	0.015	0.003	0.014	-0.026	-0.012	-0.043

**Domain 2: Sleep Scheduling and Timing**	-0.027	-0.021	-0.019	-0.041	-0.007	-0.026
Bedtime not consistent daily	-0.027	-0.019	-0.052	-0.026	0.022	-0.015
Get out of bed at inconsistent times	-0.001	-0.003	-0.013	-0.007	0.001	-0.009
Stay in bed after waking up in the morning	-0.003	0.008	-0.040	-0.052	-0.013	-0.066*
Weekend catch-up sleep (WCUS)	-0.043	-0.073*	-0.005	-0.060*	-0.060*	-0.047
Napping or resting in bed for over one hour during the day	-0.012	0.012	0.014	0.019	0.030	0.027
Lack of exposure to outdoor light during the day	-0.002	0.016	0.033	-0.056	-0.009	-0.033
Lack of regular exercise	-0.011	-0.025	-0.026	-0.023	0.005	-0.030
**Domain 3: Eating/Drinking Behaviors**	-0.015	-0.021	-0.017	-0.028	0.008	-0.049
Going to bed hungry	-0.011	-0.013	-0.004	-0.026	-0.004	-0.023
Drinking caffeinated drinks (e.g., coffee, tea, coca-cola) within the four hours prior to bedtime	-0.027	0.022	-0.017	0.016	-0.011	-0.021
Drinking alcohol within the two hours prior to bedtime	-0.008	0.038	0.045	0.049	0.023	0.030
Consuming stimulating substances (e.g., nicotine) during the two hours prior to bedtime	0.022	0.031	0.036	0.047	-0.002	0.021
Drinking a lot during the hour prior to sleep	0.014	-0.025	-0.055	-0.015	0.029	-0.013
Eating too much food during the hour prior to sleep	-0.022	-0.035	-0.003	-0.024	-0.009	-0.061*
**Domain 4: Sleep Environment**	0.027	-0.010	-0.019	0.015	0.000	-0.013
Sleep environment is either too noisy or too quiet	0.001	-0.007	-0.020	-0.008	0.004	-0.033
Sleep environment is either too bright or too dark	0.055	0.004	-0.003	0.005	0.001	-0.021
Sleep environment is either too humid or too dry	0.019	-0.013	-0.006	0.035	0.017	-0.010
Feeling too hot or too cold during sleep	0.017	-0.003	-0.027	0.047	0.004	-0.011
Poor ventilation of bedroom	-0.019	-0.034	-0.020	0.011	-0.041	0.004
Uncomfortable bedding and/or pillow	0.030	-0.014	-0.016	0.019	0.005	0.016
Too many sleep-unrelated items in bedroom	0.026	0.001	-0.013	0.031	-0.002	0.028
Sleep is interrupted by bed partner	0.043	0.020	0.029	-0.023	0.008	-0.012

Data was presented as correlation coefficient (r).

Abbreviations: *OGTT*, oral glucose tolerance test; *FBG*, fasting blood glucose; *1h BG*, 1-hour blood glucose; *2h BG*, 2-hour blood glucose; *HbA1c*, glycated hemoglobin A1c;

PSQI, the Pittsburgh Sleep Quality Index; *ESS*, the Epworth Sleepiness Scale; *SHPS*, the Sleep Hygiene Practice Scale; *WCUS*, weekend catch-up sleep.

$^{*}P < 0.05$, $^{**}P < 0.01$.
