# Supplementary material for: Sleep conditions and sleep hygiene behaviors in early pregnancy are associated with gestational diabetes mellitus: A propensity-score matched study
Source: Sleep Breath. 2024 Aug 27;28(6):2421–30. doi: 10.1007/s11325-024-03071-8 (PMC11567980; doi:10.1007/s11325-024-03071-8)
Supplement: Supplementary file 2 — Supplementary Material 2 [file 11325_2024_3071_MOESM2_ESM.pdf]

Supplementary Information (SI)

Online Resource 2

Sleep conditions and sleep hygiene behaviors in early pregnancy are associated with gestational diabetes mellitus: A propensity-score matched study

Sleep and Breathing

Guojun Ma[1,2,3, a] · Yanqing Cai[1,2,3, a] · Yong Zhang[1,2,3,*] · Jianxia Fan[1,2,3,*]

Co-corresponding author: Yong Zhang and Jianxia Fan

Address correspondence to:

Dr. Jianxia Fan, Department of Obstetrics and Gynecology, the International Peace Maternity and Child Health Hospital, School of Medicine, Shanghai Jiao Tong University, 910 Hengshan Road, Shanghai, 200030, China. Tel: +8613916212979. E-mail address: fanjianxia122@126.com

Dr. Yong Zhang, Department of Obstetrics and Gynecology, the International Peace Maternity and Child Health Hospital, School of Medicine, Shanghai Jiao Tong University, 910 Hengshan Road, Shanghai, 200030, China. Tel: +8613916472189. E-mail address: yongz415@163.com

Online Resource 2 Sleep hygiene behaviors of participants in the propensity-score matched cohort.

	Non-GDM group n=608	GDM group n=608	Z	Crude *P*-value	Adjusted *P*-value†	Adjusted OR (95%CI) †
**SHPS-Total score**	61.00(53.00,70.00)	61.00(53.00,71.00)	-0.453	0.650	0.670	1.002(0.993-1.011)
**Domain 1: Arousal-related Behaviors**	17.00(15.00,21.00)	17.00(15.00,21.00)	-0.729	0.466	0.424	1.010(0.986-1.033)
Doing sleep-irrelevant activities in bed (e.g., watching TV, reading)	4.00(2.00,5.00)	4.00(2.00,5.00)	-0.017	0.987	0.849	0.992(0.917-1.074)
Worry about not being able to fall asleep in bed	1.00(1.00,2.00)	2.00(1.00,2.00)	-2.058	0.040	0.041	1.123(1.005-1.255)
Unpleasant conversation prior to sleep	2.00(1.00,2.00)	2.00(1.00,2.00)	-0.001	>0.999	0.837	0.984(0.842-1.150)
Not enough time to relax prior to sleep	2.00(1.00,2.00)	2.00(1.00,2.00)	-0.210	0.834	0.912	1.006(0.901-1.124)
Falling asleep with TV or music on	1.00(1.00,2.00)	1.00(1.00,2.00)	-0.541	0.588	0.930	1.005(0.896-1.128)
Pondering about unresolved matters while lying in bed	2.00(2.00,3.00)	2.00(2.00,3.00)	-0.447	0.655	0.641	1.025(0.923-1.138)
Check the time in the middle of night	2.00(1.00,3.00)	2.00(1.00,3.00)	-1.022	0.307	0.390	1.042(0.948-1.146)
Worry about night-time sleep during the day	1.00(1.00,2.00)	1.00(1.00,2.00)	-1.017	0.309	0.561	1.037(0.917-1.173)
Vigorous exercise during the two hours prior to sleep	1.00(1.00,1.00)	1.00(1.00,1.00)	-0.045	0.964	0.892	0.983(0.768-1.258)
**Domain 2: Sleep Scheduling and Timing**	22.00(19.00,25.00)	22.00(19.00,26.00)	-0.420	0.675	0.696	1.005(0.981-1.029)
Bedtime not consistent daily	3.00(2.00,3.00)	3.00(2.00,3.00)	-0.029	0.977	0.986	0.999(0.884-1.129)
Get out of bed at inconsistent times	2.00(2.00,3.00)	2.00(2.00,3.00)	-1.255	0.210	0.172	1.092(0.962-1.239)
Stay in bed after waking up in the morning	3.00(2.00,4.00)	3.00(2.00,4.00)	-0.859	0.390	0.357	1.045(0.952-1.147)
Weekend catch-up sleep (WCUS)	4.00(3.00,5.00)	3.00(3.00,5.00)	-1.300	0.194	0.184	0.943(0.866-1.028)
Napping or resting in bed for over one hour during the day	3.00(2.00,4.00)	3.00(2.00,4.00)	-0.929	0.353	0.517	1.028(0.946-1.115)
Lack of exposure to outdoor light during the day	3.00(2.00,4.00)	3.00(2.00,4.00)	-0.728	0.467	0.514	1.028(0.946-1.118)
Lack of regular exercise	4.00(3.00,5.00)	4.00(3.00,5.00)	-0.353	0.724	0.723	0.985(0.904-1.072)
**Domain 3: Eating/Drinking Behaviors**	8.00(7.00,10.00)	9.00(7.00,10.00)	-0.575	0.565	0.657	1.012(0.960-1.067)

Going to bed hungry	2.00(2.00,3.00)	2.00(2.00,3.00)	-0.184	0.854	0.868	1.009(0.906-1.124)
Drinking caffeinated drinks (e.g., coffee, tea, coca-cola) within the four hours prior to bedtime	1.00(1.00,1.00)	1.00(1.00,1.00)	-0.031	0.976	0.789	1.029(0.833-1.271)
Drinking alcohol within the two hours prior to bedtime	1.00(1.00,1.00)	1.00(1.00,1.00)	-1.036	0.300	0.127	1.488(0.893-2.479)
Consuming stimulating substances (e.g., nicotine) during the two hours prior to bedtime	1.00(1.00,1.00)	1.00(1.00,1.00)	-1.900	0.057	0.376	1.607(0.562-4.592)
Drinking a lot during the hour prior to sleep	1.00(1.00,2.00)	1.00(1.00,2.00)	-0.142	0.887	0.868	1.015(0.852-1.208)
Eating too much food during the hour prior to sleep	2.00(1.00,2.00)	2.00(1.00,2.00)	-0.270	0.787	0.954	1.004(0.877-1.136)
**Domain 4: Sleep Environment**	12.00(9.00,16.00)	12.00(9.00,16.00)	-0.245	0.806	0.883	0.998(0.975-1.022)
Sleep environment is either too noisy or too quiet	2.00(1.00,2.00)	2.00(1.00,2.00)	-0.123	0.902	0.466	1.043(0.931-1.168)
Sleep environment is either too bright or too dark	1.00(1.00,2.00)	1.00(1.00,2.00)	-0.907	0.364	0.912	1.007(0.886-1.145)
Sleep environment is either too humid or too dry	1.00(1.00,2.00)	1.00(1.00,2.00)	-0.594	0.552	0.537	0.955(0.826-1.105)
Feeling too hot or too cold during sleep	1.00(1.00,2.00)	1.00(1.00,2.00)	-0.767	0.443	0.462	0.944(0.808-1.102)
Poor ventilation of bedroom	2.00(1.00,2.00)	2.00(1.00,2.00)	-0.842	0.400	0.269	0.942(0.847-1.047)
Uncomfortable bedding and/or pillow	1.00(1.00,2.00)	1.00(1.00,2.00)	-0.479	0.632	0.786	1.019(0.888-1.170)
Too many sleep-unrelated items in bedroom	1.00(1.00,2.00)	1.00(1.00,2.00)	-0.166	0.868	0.391	0.934(0.799-1.092)
Sleep is interrupted by bed partner	2.00(1.00,3.00)	2.00(1.00,3.00)	-1.354	0.176	0.252	1.062(0.958-1.177)

Data was presented as median (interquartile range, IQR).

Abbreviations: *GDM*, gestational diabetes mellitus; *SHPS*, the Sleep Hygiene Practice Scale; *WCUS*, weekend catch-up sleep; *OR*, odds ratio; *CI*, confidence interval.

† Adjusted for all baseline clinical factors mentioned in PSM.
