# Supplementary material for: Sleep conditions and sleep hygiene behaviors in early pregnancy are associated with gestational diabetes mellitus: A propensity-score matched study
Source: Sleep Breath. 2024 Aug 27;28(6):2421–30. doi: 10.1007/s11325-024-03071-8 (PMC11567980; doi:10.1007/s11325-024-03071-8)
Supplement: Supplementary file 3 — Supplementary Material 3 [file 11325_2024_3071_MOESM3_ESM.pdf]

Supplementary Information (SI)

Online Resource 1

Sleep conditions and sleep hygiene behaviors in early pregnancy are associated with gestational diabetes mellitus: A propensity-score matched study

Sleep and Breathing

Guojun Ma[1,2,3, a] · Yanqing Cai[1,2,3, a] · Yong Zhang[1,2,3,*] · Jianxia Fan[1,2,3,*]

Co-corresponding author: Yong Zhang and Jianxia Fan

Address correspondence to:

Dr. Jianxia Fan, Department of Obstetrics and Gynecology, the International Peace Maternity and Child Health Hospital, School of Medicine, Shanghai Jiao Tong University, 910 Hengshan Road, Shanghai, 200030, China. Tel: +8613916212979. E-mail address: fanjianxia122@126.com

Dr. Yong Zhang, Department of Obstetrics and Gynecology, the International Peace Maternity and Child Health Hospital, School of Medicine, Shanghai Jiao Tong University, 910 Hengshan Road, Shanghai, 200030, China. Tel: +8613916472189. E-mail address: yongz415@163.com

Online Resource 1 Sleep conditions of participants in the propensity-score matched cohort.

	Non-GDM group n=608	GDM group n=608	Z/χ^2	Crude *P*-value	Crude OR (95%CI)	Adjusted *P*-value†	Adjusted OR (95%CI) †
**Sleep Quality**							
PSQI-Total score	5.00(4.00,7.00)	6.00(4.00,8.00)	-3.828	<0.001		<0.001	1.082(1.038-1.128)
PSQI-Distribution			14.773	<0.001	1.557(1.242-1.952)		
0-5	344(56.6)	277(45.6)					1
>5	264(43.4)	331(54.4)				<0.001	1.585(1.261-1.992)
PSQI-Quality	1.00(1.00,1.00)	1.00(1.00,1.00)	-2.571	0.010		0.008	1.265(1.064-1.505)
PSQI-Latency	1.00(0.00,1.00)	1.00(0.00,2.00)	-2.946	0.003		0.005	1.212(1.059-1.386)
PSQI-Duration	0.00(0.00,1.00)	0.00(0.00,1.00)	-2.788	0.005		0.003	1.317(1.097-1.581)
PSQI-Efficiency	0.00(0.00,1.00)	0.00(0.00,1.00)	-2.901	0.004		0.003	1.236(1.073-1.423)
PSQI-Disturbance	1.00(1.00,1.00)	1.00(1.00,1.00)	-3.032	0.002		0.005	1.460(1.123-1.897)
PSQI-Medication	0.00(0.00,0.00)	0.00(0.00,0.00)	-0.498	0.618		0.733	0.912(0.537-1.548)
PSQI-Daytime dysfunction	2.00(1.00,3.00)	2.00(1.00,3.00)	-0.779	0.436		0.371	1.050(0.943-1.169)
**Alertness/sleepiness**							
ESS-Total score	5.00(3.00,8.00)	6.00(4.00,9.00)	-2.711	0.007		0.004	1.046(1.014-1.079)
ESS-Distribution			6.107	0.047		0.031	
0-6	373(61.3)	331(54.4)					1
7-10	165(2.1)	190(31.3)		0.046	1.298(1.005-1.676)	0.040	1.311(1.012-1.699)
> 10	70(11.5)	87(14.3)		0.058	1.401(0.989-1.983)	0.051	1.418(0.999-2.014)

Data was presented as median (interquartile range, IQR) or frequency (percentage).

Abbreviations: *GDM*, gestational diabetes mellitus; *PSQI*, the Pittsburgh Sleep Quality Index; *ESS*, the Epworth Sleepiness Scale; *OR*, odds ratio; *CI*, confidence interval.

† Adjusted for all baseline clinical factors mentioned in PSM.
